# Rapid access multidisciplinary lymph node diagnostic clinic: analysis of 550 patients

**DOI:** 10.1038/sj.bjc.6600738

**Published:** 2003-02-10

**Authors:** I Chau, M T Kelleher, D Cunningham, A R Norman, A Wotherspoon, P Trott, P Rhys-Evans, G Querci Della Rovere, G Brown, M Allen, J S Waters, S Haque, T Murray, L Bishop

**Affiliations:** 1 Department of Medicine, Royal Marsden Hospital, Surrey, UK; 2 Department of Computing, Royal Marsden Hospital, Surrey, UK; 3 Department of Histopathology, Royal Marsden Hospital, Surrey, UK; 4 Department of Cytology, Royal Marsden Hospital, Surrey, UK; 5 Head and Neck Unit, Royal Marsden Hospital, Surrey, UK; 6 Department of Surgery, Royal Marsden Hospital, Surrey, UK; 7 Department of Diagnostic Imaging, Royal Marsden Hospital, London, UK

**Keywords:** lymphadenopathy, malignancy, diagnosis, fine-needle aspiration, ultrasound

## Abstract

Lymphadenopathy is common, affecting patients of all ages. The current referral pattern for investigating patients with lymphadenopathy varies widely with no universally practised pathway. Our institution set up a lymph node diagnostic clinic (LNDC) accepting direct referrals from primary care physicians. Details of clinical presentation and investigations were recorded prospectively. Between December 1996 and July 2001, 550 patients were referred (M: 203; F:347). The median age was 40 years (range 14–90). The median time between initial referral and the first clinic visit was 6 days. Of 95 patients diagnosed to have malignant diseases, the median time from the first clinic visit to reaching malignant diagnosis was 15 days. Multivariate logistic regression analysis identified five significant predictors for malignant nodes: male gender (risk ratio (RR)=2.72; 95% confidence interval (CI): 1.63–4.56), increasing age (RR=1.05; 95% CI: 1.04–1.07), white ethnicity (RR=3.01; 95% CI: 1.19–7.6) and sites of lymph nodes: supraclavicular region (RR=3.72; 95% CI: 1.52–9.12) and ⩾2 regions of lymph nodes (RR=6.41; 95% CI: 2.82–14.58). Ultrasound and fine-needle aspiration cytology of palpable lymph nodes were performed in 154 and 289 patients, respectively. An accuracy of 97 and 84% was found, respectively. In conclusion, a multidisciplinary lymph node diagnostic clinic enables a rapid, concerted approach to a common medical problem and patients with malignant diseases were diagnosed in a timely fashion.

Lymphadenopathy (LA) is common and affects patients of all ages. An annual incidence of 0.6–0.7% has been estimated for the general population (Allhiser *et al*, 1981; Fijten and Blijham, 1988). Patients with lymphadenopathy present to a wide range of medical specialties. When physicians are faced with these patients, the critical tasks are to differentiate benign from malignant lymph nodes, to identify serious medical conditions that require specialist care, and to reassure patients with benign reactive lymphadenopathy or self-limiting diseases.

The possibility of malignancy raises the most concern among patients and health care professionals. Prompt referral for definitive investigations and treatment cannot be overstated, as cancer is perhaps the disease people fear most. Despite the incidences of many cancers being broadly similar in England compared with other European countries and the USA, the corresponding survival rates are poorer (Table 1

**Table 1:** Five-year relative survival of common cancers

	**Breast cancer (%)**	**Lung cancer (%)**	**Colorectal cancer (%)**	**Non-Hodgkin's lymphoma (%)**	**Hodgkin's disease (%)**
England and Wales	68	6	39	45	75
Europe	73	11	55	48	75
USA	86	14.5	61	53	83

Data adapted from: SEER Cancer Statistic Review, 1973–1998; Cancer Survival Trends in England and Wales 1971–1995.

) (Office for National Statistics, 1999; Ries *et al*, 2001). To address this problem, the British government proposed, in September 2000, the National Health Service (NHS) Cancer Plan to provide a comprehensive strategy for bringing together prevention, screening, diagnosis, treatment and care for cancer to improve survival (Department of Health, 2000b). The current guideline recommends all patients with lymphadenopathy of more than 1 cm in size persisting for 6 weeks to be referred urgently for further evaluation (Department of Health, 2000a).

Apart from cancer, there are also other medical conditions presenting with LA that will require urgent medical attention. This would include infections such as tuberculosis (TB) and human immunodeficiency virus (HIV), both of which pose an important public health problem. In addition, immune-induced injury disorders such as systemic lupus erythematosus, sarcoidosis and rheumatoid arthritis will also require specialist care.

Even after the general practitioner (GP) has decided to refer a patient with lymphadenopathy for definitive diagnostic assessment, the referral pattern to specialists varies at the present time. Some patients may be referred to general surgeons for biopsy, some to haematologists for evaluation and others to various specialties depending on sites of LA and associated clinical features. In some cases, this could lead to delays in diagnosis, resulting in increased anxiety among patients and further postponement in commencing definitive treatment.

Royal Marsden Hospital (RMH) is a tertiary referral comprehensive cancer centre. Patients are normally referred from secondary referral hospitals after a diagnosis of cancer has been made. However, in an attempt to overcome the aforementioned problems in the referral pathway for patients with LA, a lymph node diagnostic clinic (LNDC) was set up at our institution. The primary aim was to reach rapid diagnosis in patients with LA using a concerted multidisciplinary approach. Preliminary results on the first year's activity of this clinic have been reported previously (Gregory *et al*, 2000). This paper reports on the first consecutive 550 patients referred between 1996 and 2001 to our lymph node diagnostic clinic.

## SETTING AND METHOD

Royal Marsden Hospital serves a local population of 2.2 million residents. General practitioners (GPs) from 151 practices within five local health authority regions referred patients to the LNDC. Table 2

**Table 2:** Demographic characteristics of population

		**Sex (%)**		**Age (%)**		**Ethnicity (%)**					**Social classes (%)**		
**Population within each local health authority**	**Number in population**	**Male**	**Female**	**18–44**	**⩾45**	**White**	**Black (African and Carribean)**	**Asian (Indian, Pakistani, Bangladeshi)**	**Chinese**	**Others**	**I–II**	**III–V**	**Not classified**
MSW	687 449	49	51	49	49	87	4	4	1	4	30	50	20
Croydon	338 000	49	51	49	49	82	7	6	1	4	34	51	15
East Surrey	418 364	49	51	49	49	96	<0.5	2	1	1	40	48	13
KR	334 032	49	51	49	49	94	<0.5	3	1	2	41	44	15
CWK	400 000	49	51	49	49	83	5	4	1	7	45	36	19
All England average	49 495 000	49	51	49	49	94	2	3	<0.5	1	27	55	17
Our study	550	37	63	60	40	82	6	4	1	3	NC	NC	NC

Local Health Authorities: MSW=Merton, Sutton and Wandsworth; CWK=Chelsea, Westminster and Kensington; KR=Kingston and Richmond; NC=data not collected. As figures are rounded down, percentages do not necessarily add up to 100.

shows the demographics of the local population. The LNDC was set up to run alongside existing clinics. Effectively this service was provided 4 days per week. Invitation letters were sent to GPs who had other cancer patients under follow-up at our institution. Referrals were accepted directly from GPs for patients with unexplained lymphadenopathy. Aside from lymphadenopathy, some patients were referred for ‘lumps’ in extranodal sites.

Patients were initially assessed by a medical team comprising medical oncologists and research nurses. The lead clinician (DC) supervised the operation of the clinic during the entire study period. According to clinical presentations, the following investigations were carried out as appropriate: haematological assays including complete blood count, erythrocyte sedimentation rate and peripheral blood film; biochemistry profiles including serum electrolytes, urea, creatinine, bilirubin, total protein, serum albumin, liver transaminases and alkaline phosphatases, lactate dehydrogenase (LDH), immunoglobulins, *β*_2_ microglobulin, plasma and urine electrophoresis; bacterial, viral and parasitic serology and culture; imaging including chest X-ray (CXR), ultrasound (US), computed tomography (CT) and magnetic resonance imaging (MRI); fine-needle aspiration and lymph node biopsy. A written guideline for investigations was available in the Royal Marsden Hospital Lymphoma Unit Clinical Guidelines (Protocols and Policies) document.

Fine-needle aspirations were carried out on clinically suspicious malignant lymph nodes. Fine-needle aspiration cytology (FNAC) findings were graded from C0 to C5 according to traditional cytomorphological methods (Trott, 1985). Definitions of C0–C5 are shown in Table 8

**Table 8:** Fine-needle aspiration cytology (FNAC)

	**Number (*n*=289)**		
**FNAC**	**Benign tumours**	**Malignant tumours**	**Nonmalignant disease**
C0	4	4	22
C1			10
C2	14	35	155
C3		1	5
C4		14	
C5	1	23	1

C0 denotes a sample as insufficient, occasionally containing no cells at all. C1 has scant normal cells present that are too few to characterise. C2 contains normal cells that are not suspicious for malignancy. C3 displays cells, which, though suspicious, are not classifiable. C4 indicates a high probability of malignancy with inconclusive subtype. C5 is diagnostic of a particular specified malignancy.

. For lymph nodes that were clinically very suspicious or cytological grades C3–C5, a formal lymph node biopsy would be performed after appropriate surgical assessment. Exceptions were cases where FNAC was diagnostic of carcinoma (C5) and primary sites were readily identified. Lymph node biopsies were carried out by head and neck, breast, plastic or general surgeons, depending on sites of lymphadenopathy.

All patients returned to the LNDC to receive the results of investigations. Patients diagnosed to have lymphoma were managed within the lymphoma unit. Patients with malignancies other than lymphoma were referred internally to other appropriate units. Patients with non-neoplastic diagnoses that required specific medical treatment were referred to the appropriate specialty at another hospital. Other patients with benign reactive lymphadenopathy or self-limiting diseases were reassured and discharged from the LNDC. On some occasions, several clinic visits at variable time intervals were required to ensure resolution of symptoms and lymphadenopathy before formal discharge from the LNDC. All discharged patients were advised to return to the LNDC if the lymph node(s) enlarged or there was appearance of new lymph node(s).

Details of patients' demographics, clinical presentations, laboratory and imaging results, final diagnosis and subsequent management were collected prospectively in the lymphoma research databases. In addition, all case notes and investigation results from all patients were available in our hospital electronic patient record (EPR) system. To ensure high-quality data collection, all patients' data recorded in the database in this series were cross-referenced with EPR by two of the investigators (MK and IC) independently. Discrepancies were verified with patients' written notes if necessary and the database was appended accordingly. A single cytopathologist (PT) reviewed all FNAC, and a single histopathologist (AW) reviewed all lymph node biopsies where a final diagnosis of lymphoma was made.

## STATISTICAL CONSIDERATIONS

Descriptive statistics were used for the epidemiology of lymphadenopathy in this series. The date of malignant diagnosis was defined as the date of FNAC reporting in carcinoma and histology reporting in lymphoma. The time from initial referral to date of first definitive treatment (either chemotherapy or radiotherapy) was determined for Hodgkin's disease and diffuse large B-cell non-Hodgkin's lymphoma (NHL) as these are the two large subgroups of patients who would require immediate treatment.

Stepwise logistic regression models were constructed using a binary outcome specification for the presence or absence of malignancy in lymph nodes. The independent variables included in the analysis were age, gender, ethnicity, site and number of regions of lymph nodes and whether LA had a bilateral distribution. Number, size and texture of lymph nodes were subject to considerable interobserver variation and therefore not used as predictive factors in the multivariate models. Age was used as a continuous variable. Ethnicity was divided into white or non-white categories. Sites of presenting peripheral lymph nodes were grouped into cervical, supraclavicular, axillary, inguinal or ⩾2 regions of lymph nodes. Since cervical lymphadenopathy was the most common site of lymph nodes in this series, multivariate adjusted risk ratios (RR) were estimated with the RR for cervical region set at 1. The relative importance of predictive factors was measured by the Wald test with each predictive factor in the stepwise logistic regression model. The threshold for inclusion in the multivariate model was set at *P*<0.05.

Sensitivity, specificity, positive and negative predictive values and accuracy of ultrasound and FNAC as tools for diagnosing malignancy were determined. Accuracy was defined as the proportions of true positives and true negatives in the total number of investigations performed.

Data analysis was performed using SPSS version 10.1.4 (SPSS Inc., Chicago, IL, USA). All analyses were carried out in October 2001 on an ‘intention to assess’ basis.

## RESULTS

**Figure 1:**
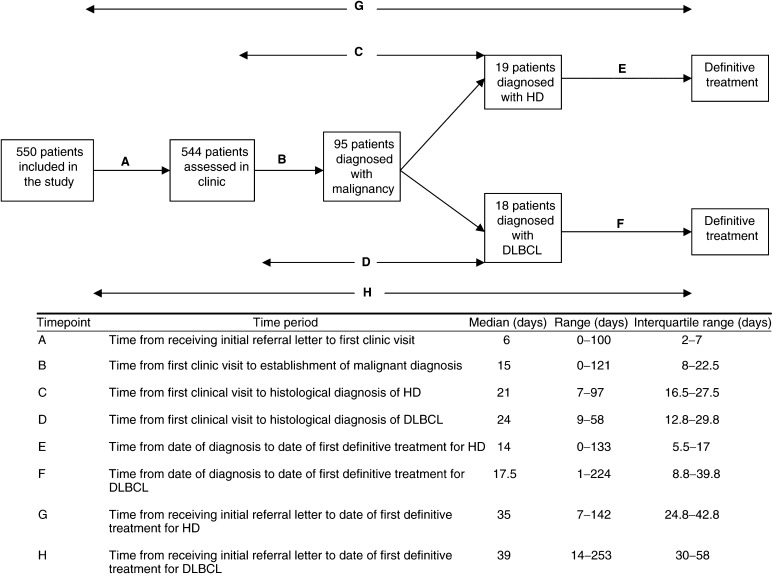
Time period from referral to diagnosis for the whole study cohort and treatment for Hodgkin's disease (HD) and diffuse large B-cell non-Hodgkin's lymphoma (DLBCL). Median, range and interquantile range are quoted. As median values are quoted, time point G does not equal the sum of the median values of A and C and E. Similarly, time point H does not equal the sum of the median values of time points A and D and F.

Between December 1996 and July 2001, 550 patients were referred to the lymph node diagnostic clinic. Table 2 shows the baseline demographics of the patients referred. The median age was 40 years (range 14–90). Each general practice referred a median of two patients (range 1–25). Figure 1

shows the time between receipt of initial referral letter and first clinic visit. The median time for this period was 6 days (including weekends and public holidays). In total, 413 patients (75.1%) were seen within 1 week of referral and 531 (96.5%) were seen within 2 weeks. All patients were offered a clinic appointment within 2 weeks. Those patients who were seen more than 2 weeks from referral did not attend their first offered clinic appointment. Six patients failed to attend their appointments. One patient attended the initial visit, but no follow-up data were available.

Ninety-five patients (17.3%) were found to have malignant disease (Table 3

**Table 3:** Diagnoses of neoplasm

**Diagnosis**	**Number**
Lymphoproliferative disorders	*n*=62
Hodgkin's disease	19
Diffuse large B-cell lymphoma	18
Follicular lymphoma	10
B-chronic lymphocytic leukaemia	4
Mantel cell lymphoma	3
T-cell lymphoma	3
Small lymphocytic lymphoma	3
Post-transplant lymphoproliferative disorder	1
Lymphoma unknown subtype	1
	
Metastatic tumours	*n*=29
Head and neck squamous cell carcinoma	10
Squamous cell carcinoma of oesophagus	1
Breast	3
Melanoma	3
Prostate	2
Nonsmall cell carcinoma of lung	2
Small cell carcinoma of lung	2
Thyroid	2
Seminoma	1
Unknown primary (two squamous cells, one small cell)	3
	
Other malignant tumours	*n*=4
Myofibroblastic tumour	1
Myeloproliferative disease	1
Sarcoma	1
Unknown	1
	
Benign tumours	*n*=21
Pleomorphic adenoma	10
Warthin's adenolymphoma	4
Schwannoma	3
Thyroid adenoma	3
Carotid body tumour	1

). The median time from the first clinic visit to establishment of malignant diagnosis was 15 days (Figure 1). Ninety-eight per cent of patients received their malignant diagnosis within 2 months. Only two patients fell outside this time period. The first patient had a delay in lymph node biopsy because of social reasons, and in the second patient the reason for the delay in biopsy was unknown.

Of the 19 patients diagnosed with Hodgkin's disease, 18 received induction chemotherapy and one received single modality radiotherapy. One patient received chemotherapy abroad after diagnosis, and therefore the date of first treatment could not be determined. The median time between date of diagnosis and date of first definitive treatment (chemotherapy or radiotherapy) was 14 days (Figure 1). Of the 18 patients diagnosed with diffuse large B-cell lymphoma, 17 patients received induction chemotherapy and one received single modality radiotherapy. The median time between date of diagnosis and date of first definitive treatment (chemotherapy or radiotherapy) was 17.5 days (Figure 1).

Twenty-one patients had benign tumours confirmed on histology (Table 3). One hundred and thirty-nine patients had non-neoplastic disease with a recognised clinical diagnosis as the cause of their lymphadenopathy or ‘lumps’ (Table 4

**Table 4:** Miscellaneous non-neoplastic diseases

**Diagnosis**	**Number (total *n*=139)**
Infections	*n*=47
Bacterial infections	Total=19
Tuberculosis	12
Streptococcus	2
Corynebacterium	1
Moxarella	1
Bartonella	3
Viral infections	Total=11
Human immunodeficiency virus	4
Epstein–Barr virus	5
Cytomegalovirus	1
Hepatitis C	1
Fungal/protozoal/parasitic infestations	Total=17
Toxoplasmosis	15
Pediculosis/dermatophytosis	2
Immune-mediated injury disorders	Total=13
Lupus erythematosus	6
Sarcoidosis	6
Rheumatoid arthritis	1
Primary skin diseases	Total=5
Others	Total=73

). Figure 2

**Figure 2:**
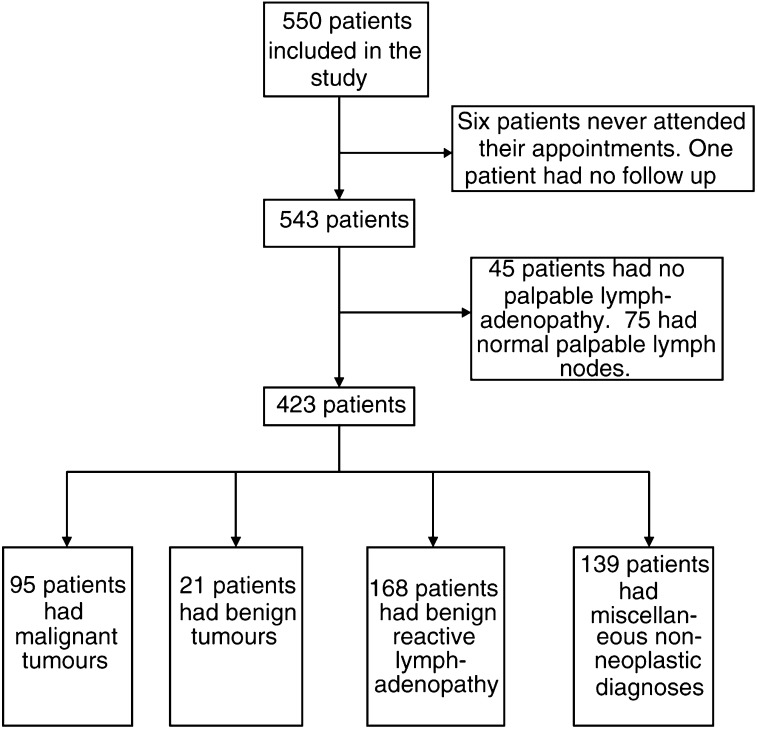
Diagnostic pathway for the whole study cohort.

summarises the diagnostic pathway of all patients.

Table 5

**Table 5:** Presenting lymph node regions

**Lymph node regions**	**Number of patients Total *n*=550 (%)**	**Number of patients with malignant diseases^a^ Total *n*=95 (%)**
Head and neck (level I–III and V)	254 (46.2)	35 (13.8)
Supraclavicular (level IV)	35 (6.4)	12 (34.3)
Axilla	53 (9.6)	8 (15.1)
Inguinal	41 (7.5)	7 (17.1)
⩾2 regions	87 (15.8)	30 (34.5)
Extranodal^b^	80 (14.5)	3 (3.8)

a Malignancy refers to presenting lymph node regions in patients with proven malignancy. Percentage refers to the number of malignant nodes over the total number of patients with lymph nodes in a particular region. For example: for head and neck, 35 out of 254 patients gives 13.8%.

b Extranodal denotes ‘lumps’ not present in the peripheral palpable lymph node regions.

shows the distribution of the presenting lymph node sites for the whole cohort. Eighty patients (14.5%) had ‘lumps’ in extranodal sites. These lumps included thyroid, cutaneous, facial and breast lumps.

When multivariate logistic regression analysis was performed in our cohort, the following factors were highly significant predictors for malignant lymphadenopathy: male gender, increasing age, white ethnicity, supraclavicular lymph nodes and ⩾2 regions of lymph nodes (Table 6

**Table 6:** Multivariate logistic regression model predicting malignancy in patients with lymphadenopathy (LA)

			**95% confidence interval**	
**Predictive factors**	**Significance**	**Risk ratio**	**Lower limit**	**Upper limit**
Age	*P*<0.001	1.05	1.04	1.07
Male sex	*P*<0.001	2.72	1.63	4.56
Ethnicity–White	*P*=0.02	3.01	1.19	7.6
Supraclavicular fossa LA	*P*=0.004	3.72	1.52	9.12
⩾2 regions of LA	*P*<0.001	6.41	2.82	14.58
Extranodal sites	*P*=0.03	0.24	0.07	0.83

). Extranodal ‘lumps’ had significantly low risk for malignancy. For each 10-year increase in age, the increase in risk of malignancy was 1.64 (95% CI 1.44–1.88). This model has a sensitivity of 30.5%, a specificity of 97.1% and an accuracy of 85.6%.

Ultrasound (US) scans of clinically suspicious lymphadenopathy were performed in 154 patients (28%). One hundred and three patients had normal US findings and all of them had nonmalignant diseases. Abnormal US findings were seen in 51 patients. These findings correctly identified five benign tumours, 11 malignant tumours and 30 non-neoplastic causes of LA. Ultrasound incorrectly raised suspicion of malignancy in only five patients. In our series, US as a diagnostic tool for detecting malignancy gave an accuracy of 97% (Table 7

**Table 7:** Accuracy of investigations to detect malignancy

**Tests**	**No.**	**Sensitivity (%)**	**Specificity (%)**	**Positive predictive value (%)**	**Negative predictive value (%)**	**Accuracy (%)**
Ultrasound	154	100	97	69	100	97
Fine-needle aspiration	289	49	97	84	84	84

).

CT scans were carried out in 132 patients in which 107 were abnormal. However, CT scans were often carried out as part of the staging in patients who had been diagnosed with malignancies rather than as primary diagnostic investigations.

Fine-needle aspiration cytology of palpable lymph nodes was performed in 289 patients (52.5%) (Table 8). Fifteen patients with lymphadenopathy highly suspicious of malignancy were referred directly for lymph node biopsy. Seventy-seven out of 95 patients (81.1%) with malignancies had fine-needle aspirations performed. Within this group of 77 patients, 38 (49.3%) had FNAC reported as between C3 and C5, but 35 (45%) had FNAC reported to show no malignant cells (C2). In this latter group with false-negative results, 83% had lymphoid malignancies. Two patients had FNAC reported as C5, but turned out to have benign conditions after excision. The first was a 39-year-old male smoker with parotid swelling. Cytology was reported as probable salivary adenoid cystic carcinoma. Subsequent superficial parotidectomy revealed a pleomorphic adenoma. The second patient with submandibular swelling revealed reactive lymph nodes with no evidence of malignancy after selective neck dissection.

Two hundred and ten patients with nonmalignant processes had FNAC performed. Five patients had FNAC showing C3 and proceeded to formal excision biopsy. Two displayed reactive lymphadenopathy of unknown cause. Two patients had acute toxoplasmosis with concordant changes on biopsy. One patient had caseating granulomata on biopsy and tuberculosis was confirmed. In our series, FNAC as a diagnostic tool for detecting malignancy gave an accuracy of 84% (Table 7).

Two hundred and seven patients (37.6%) were referred for lymph node biopsy. Excision biopsies were performed in 182 cases. One patient declined biopsy and 24 patients did not proceed to biopsy after referral. The latter was in general because of resolution of lymphadenopathy between referral and biopsy dates. Eighty-seven patients with malignancy had formal tissue biopsy performed. For those who did not have formal histology, three were diagnosed with FNAC of C5 and tumour masses were seen in lung or thyroid on imaging. Two patients were diagnosed on bone marrow biopsy. One elderly woman suffered from severe comorbid conditions precluding further investigations. One patient had a diagnosis of lymphoma made clinically in another hospital after refusing further investigations at our institution. One patient chose to seek alternative therapy from a religious group. At autopsy, a diagnosis of sarcoma was confirmed.

Fifty-two out of 120 patients (43%) with normal/absent lymph nodes and 16 out of 168 patients (10%) with benign reactive lymphadenopathy were discharged from the clinic on their initial visits after reassurance. The remaining patients with normal/absent lymph nodes or benign reactive lymphadenopathy underwent a period of observation before formal discharge.

## DISCUSSION

A comprehensive review of the epidemiology and diagnostic work-up of 550 patients referred to our lymph node diagnostic clinic was undertaken. Patients were assessed in a timely fashion with a median time of 6 days between receipt of the referral letter and the first clinic visit, and all patients were offered an appointment within 2 weeks. Malignancies were diagnosed with a median time of 15 days from the patients' initial visits, and those with potentially curable cancers such as Hodgkin's disease and diffuse large B-cell lymphoma completed their staging investigations and commenced their definitive treatment in just over 2 weeks after diagnosis.

The poorer cancer survival in England compared to other European countries and the USA has been attributed to the more advanced stage of disease by the time patients are treated and the prolonged time taken in hospitals to progress from the first appointment through diagnostic tests to treatment (Department of Health, 2000b). A multidisciplinary lymph node diagnostic clinic therefore has the advantage of concentrating expertise, facilitating investigations and ensuring prompt commencement of appropriate treatment. In particular, considering the diversity of causes of lymphadenopathy, patients with malignant disease in our series were able to reach their diagnosis in just over 2 weeks. No other studies investigating lymphadenopathy have reported this timing. A GP in England, with an average list size of 2000 patients, will only see one or two new patients with lung cancer, one with breast cancer and one with colorectal cancer per annum. For NHL, a GP may see one case every 4 years, and with Hodgkin's disease, one case every 20 years (Department of Health, 2000a). The relatively few cases seen by individual GPs makes it difficult to identify those patients at highest risk.

The NHS Cancer Plan set milestones for all patients to be treated within 2 months of urgent referral and within 1 month of diagnosis by 2005 (Department of Health, 2000b). These targets were achieved in our series with HD and diffuse large cell NHL. The median time between initial referral and first treatment was just over 7 weeks in our series. This encompassed the time required for diagnosis, imaging for staging, lymph node and bone marrow biopsy and histological interpretations. In our series, this time period was not determined for other lymphoma subtypes and solid tumours as a wide spectrum of treatment approaches are possible. Sometimes an observation period may be adopted in the case of follicular lymphoma and chronic lymphocytic leukaemia. One other study has reported this time period between initial referral and first treatment in lymphoma (Summerfield *et al*, 2000). Eighty-eight patients presented between 1997 and 1999 to three institutions in the UK were assessed. The average delay from GP referral to hospital appointment was 3.9 weeks. The average diagnostic delay was 2.8 months and treatment delay was 1.2 months. Thus, an average of more than 21 weeks was required for this cohort of patients to progress from initial referral to first treatment. This time period was three times longer than that achieved with our LNDC.

Nevertheless, a time period of 7 weeks from initial referral to first definitive treatment still leaves room for improvement. A retrospective national survey in the UK showed that apart from breast and ovarian cancer, no other major cancer type patients received first definitive treatment within a median of 7 weeks even when the referral was urgent (Spurgeon *et al*, 2000). A recent study in Canada showed that the median waiting time for 1456 patients undergoing cancer surgery was 37 days and 37.2% of patients were judged by their surgeons to have had an inappropriate wait (Simunovic *et al*, 2001). A study in patients with lung cancer has reported that only 32.4% of patients received definitive treatment within 8 weeks of the first hospital visit (Melling *et al*, 2002) in comparison with 75% of patients with diffuse large B-cell NHL and 95% of patients with HD in our series. In two other studies in Canadian breast cancer patients, it was found that the median time from screening examination to diagnosis was 3.7 weeks (Olivotto *et al*, 2001) and the median time from diagnosis to breast cancer surgery was 4.9 weeks (Mayo *et al*, 2001). However, patients in these studies were already identified as suspected malignancies, and were therefore not comparable to those presented to our LNDC who were referred to evaluate lymphadenopathy of unknown aetiology not necessarily of malignant origin.

The impact of delay in diagnosis and treatment on survival is a controversial area. A systematic review in breast cancer showed that delays of 3 months or more had 12% worse 5-year survival compared to those with shorter delays (Richards *et al*, 1999), although another large study did not find such an association and was not included in the systematic review (Sainsbury *et al*, 1999). Indeed, this latter study found that patients with a delay of less than 30 days between GP referral and treatment had worse survival than patients in any other time period, a paradoxical finding also illustrated in endometrial cancer (Crawford *et al*, 2002). However, this may reflect the cancer biology – a more aggressive clinical picture would prompt higher suspicion for malignancy and a shorter starting time for the treatment of more advanced disease which has a worse outcome. Other studies evaluating diagnostic and treatment delays in other cancers and survival were inconclusive, but they suffered from small sample sizes, and therefore could not detect small but clinically meaningful survival differences (Norum, 1995; Roncoroni *et al*, 1999; Bozcuk and Martin, 2001; Jensen *et al*, 2002). Assessing the impact of delay in diagnosis and treatment on survival will therefore require continuing research effort.

In the primary care setting, the prevalence of malignancy in patients with LA was between 0 and 1.3% (Allhiser *et al*, 1981; Williamson Jr, 1985; Fijten and Blijham, 1988). Our malignancy pick-up rate of 17.3% was, however, similar to that found in another series from Greece (Vassilakopoulos and Pangalis, 2000). Other solid tumour one-stop clinics reported malignancy pick-up rates of 3–10% (Gui *et al*, 1995; Berry *et al*, 1998; Toomey *et al*, 1998; Lamah *et al*, 2000; Patel *et al*, 2000). Of note, relative to other lymphomas, a higher than anticipated incidence of Hodgkin's disease was seen in our series. All but one had nodular sclerosing histological subtype and the majority of patients were young adults. Our LNDC provided a gateway whereby young adults with this highly curable malignancy could be swiftly diagnosed.

Our multivariate logistic regression model identified increasing age, male gender, white ethnicity and site of lymph nodes as independent predictive factors for malignancy. Age has previously been recognised as the most important factor in predicting whether LA is due to a benign or malignant process (Lee *et al*, 1980). When one considers malignancies presenting with peripheral lymphadenopathy, there is a male predominance. Females are also more likely to consult primary care physicians and use diagnostic services compared to males (Bertakis *et al*, 2000) although the complaint may not necessarily be of a sinister nature. This may partly explain why males have an increased risk for malignancy in our series. Supraclavicular and generalised lymphadenopathy were two other significant predictors for malignancy in our series. Although supraclavicular lymph nodes were an infrequent finding in our cohort, they are made up of the highest proportion of malignant nodes. In a large series of supraclavicular fossa swelling or lymph node fine-needle aspirations, 55% of 309 aspirates were found to be malignant (Ellison *et al*, 1999). The presence of supraclavicular and generalised lymphadenopathy should therefore alert the clinician to the possibility of malignancy.

An algorithm for evaluating a patient with lymphadenopathy has been proposed by Greenfield and Jordan (1978), although this has been criticised as impractical, time-consuming and expensive in the primary care setting (Williamson Jr, 1985). A less aggressive approach making use of an adequate observation period has been suggested (Williamson Jr, 1985). Two groups have reported decision-making models that would identify more precisely those patients who should have a biopsy (Slap *et al*, 1986; Vassilakopoulos and Pangalis, 2000). The study by Vassilakopoulos *et al* evaluated 475 patients with lymphadenopathy in a haematology unit in Greece (Vassilakopoulos and Pangalis, 2000). A mathematical model was developed using six variables – lymph node size, location (supraclavicular or nonsupraclavicular), age (>40 years or ⩽40 years), texture (soft/semihard or hard), tenderness and generalised pruritus. Ninety-six per cent of those who required biopsy were correctly classified by this model.

It has been accepted that cross-sectional imaging has a higher accuracy than palpation in the diagnosis of neoplastic lymphadenopathy. The relative accuracy of each modality, however, is an area of continuing study (Kaji *et al*, 1997). Ultrasound evaluation is best suited for examining superficial lymph nodes because it is inexpensive, easy to perform, has no ionising radiation and can guide fine-needle aspiration at the time of examination. In addition, the recent development of Doppler sonography technology allows assessment of changes in nodal blood flow in order to differentiate metastatic from nonmetastatic nodes (Wang *et al*, 2001). A recent study showed that a combination of grey-scale and power Doppler sonography assessing internal architecture of the node may be superior to CT in differentiating metastatic from nonmetastatic nodes in the neck (Sumi *et al*, 2001). In our series, US examination had 100% sensitivity and 96% specificity for malignant nodes.

Fine-needle aspiration cytology has been accepted as a rapid, minimally invasive and accurate method for the initial evaluation of LA. Although the accuracy of diagnosing metastatic carcinoma in lymph nodes by fine-needle aspirations is in excess of 90% (Pangalis *et al*, 1993; Steel *et al*, 1995; Cha and Goates, 1996; Prasad *et al*, 1996; Nasuti *et al*, 2000), the accuracy of diagnosing primary lymphoma by fine-needle aspirations is only about 72% (Steel *et al*, 1995). However, with ancillary studies such as immunocytochemical phenotyping and/or flow cytometry, the accuracy of diagnosing haematopoietic conditions has been improved considerably (Nasuti *et al*, 2000; Liu *et al*, 2001). In our series, a false-negative rate of 13.5% in FNAC was seen, mostly in lymphoid malignancies. A false-positive rate of 2.4% in our series for malignancy was high compared with other studies (0.2–0.9%) (Steel *et al*, 1995; Nasuti *et al*, 2000). However, the proportion of nondiagnostic specimens of 10.4% was similar to another study (Nasuti *et al*, 2000). As fine-needle aspirations are operator-dependent, this shortfall in our FNAC accuracy could be partly explained by the lack of dedicated cytopathologists performing fine-needle aspirations at our clinic. Many studies had experienced cytopathologists at the clinic to provide immediate assessment. Adequate sampling and/or triage for further studies could thus be ensured. Many of these series, however, had a significant patient selection bias consisting entirely of patients with malignant lesions (Steel *et al*, 1995; Nasuti *et al*, 2000; Liu *et al*, 2001); therefore, results were strictly not comparable with ours.

The limitation of our series stems from the patients referred. Although GPs were encouraged to refer any cases of unexplained lymphadenopathy, there may be an inherent selection bias in the referral pattern depending on suspicion on malignancy. In addition, an attempt was not made to determine the differences in the time required for the diagnosis of patients referred outside the LNDC pathway and those diagnosed in our LNDC. However, such a comparison is problematic as firstly there might be different referral behaviour across different time periods because of, for example, the government initiative of the ‘2-week rule’ for urgent referral (Department of Health, 2000a). As the median number of patients referred per GP was only two, the consistency in the referral pattern across different time periods was therefore not assessable. Secondly, there may be an inherent bias of why a GP chose a particular route of referral. A randomised study of a conventional referral pathway and LNDC would be desirable but difficult to conduct as there is no consistency in the conventional referral pathway.

In conclusion, a multidisciplinary lymph node diagnostic clinic enables a rapid, concerted approach to evaluate a common medical problem. Patients with malignant diseases were able to receive their diagnoses in a timely fashion. Our clinic provides a diagnostic service delivery model to which one could compare future health service innovations. Further research is essential to integrate diagnostic services with staging investigations and treatment delivery, and this may in future lead to improved survival in these patients.
